# Case Report: A novel missense variant in *ZC4H2*, c.196C>T p.(Leu66Phe), is associated with a mild, ZC4H2-related X-linked syndromic intellectual disability (ZARD) phenotype

**DOI:** 10.3389/fped.2025.1518782

**Published:** 2025-04-10

**Authors:** Ria Garg, Wenying Zhang, Julianne E. Hartmann, Anne Slavotinek

**Affiliations:** ^1^ Center for Development, Behavior, and Genetics, State University of New York Upstate Medical University, Syracuse, NY, United States; ^2^ Department of Pediatrics, Upstate Golisano Children’s Hospital, Syracuse, NY, United States; ^3^ Department of Pediatrics, Cincinnati Children’s Hospital Medical Center, Cincinnati, OH, United States; ^4^ Department of Pediatrics, University of Cincinnati College of Medicine, Cincinnati, OH, United States

**Keywords:** contracture, neurodevelopmental disorders, growth and development, ZC4H2, ZARD

## Abstract

*ZC4H2* is an X-linked gene that has emerged as critical for neural development, synaptic functioning, and gene regulation. We present an 11-month-old male who was evaluated for bilateral congenital vertical talus identified in the newborn period. Exome sequencing identified a hemizygous, missense variant in *ZC4H2*, NM_018684.4:c.196C>T p.(Leu66Phe), that affects the same amino acid residue as a previously reported, pathogenic *ZC4H2* variant, c.197T>A p.(Leu66His). The variant was inherited from his mother, who had camptodactyly of the fifth fingers, and was also present in the maternal uncle who carried a diagnosis of cerebral palsy. The pathogenic missense variant in this family is located in the coiled-coil domain of the ZC4H2 protein. Although data remain scarce, missense variants in this domain may be associated with a milder, ZC4H2-associated rare disorder (ZARD) phenotype.

## Introduction

Pathogenic variants in the *ZC4H2* gene have been associated with Wieacker–Wolff syndrome, now called ZC4H2-related X-linked syndromic intellectual disability, or ZARD (1). The first description of ZARD reported six affected males from three generations of the same family who had congenital contractures of the feet, slowly progressive distal muscle atrophy, ocular dyspraxia, facial weakness, and intellectual disability (2). The males were all later shown to have a missense variant, c.187G>C p.(Val63Leu), that affected a highly conserved residue in the coiled-coil domain (Ncoils domains; amino acid residues 14–65 and 73–100; Ensembl website) of *ZC4H2* (3). The pathogenicity of this variant was supported by Western blot analysis, which showed that the p.(Val63Leu) variant had weaker SMAD-stabilizing activity compared to wild-type ZC4H2 in HEK293 cells (4). ZARD is now known to be a phenotypically variable, X-linked neurodevelopmental disorder that affects the central and peripheral nervous systems (2–12). The condition is characterized by arthrogryposis, developmental delays, hypotonia, feeding difficulties with poor growth, skeletal abnormalities, and facial anomalies (12). We report an additional family with relatively mildly affected individuals with ZARD who have a missense variant affecting the coiled-coil domain to contribute to the phenotypic and genotypic information associated with pathogenic missense variants in this ZC4H2 domain.

## Case report

Written informed consent was obtained for the publication of clinical data. The proband underwent genetic evaluation at 3 months of age because of bilateral congenital vertical talus (CVT), feeding difficulties, and low tone. He was a male infant born to nonconsanguineous Caucasian parents. Pregnancy was complicated by nausea and maternal hypertension requiring two weeks of bed rest. Fetal movements were reported to be reduced from 35 weeks of gestation, and non-stress tests were performed twice weekly to monitor fetal movement. Prenatal ultrasound scans displayed no structural abnormalities, and CVT was not noted. Labor was induced at 37 weeks due to reduced fetal movements and maternal hypertension, and a male infant was delivered vaginally with a weight of 2,897 g (15th centile; *Z* score −1.04), length of 47.6 cm (11th centile; *Z* score −1.21), and an occipitofrontal circumference (OFC) of 34.3 cm (45th centile; *Z* score −0.13). The neonatal period was complicated by poor sucking and swallowing and hypotonia. He had a thick lip tie that complicated feeding and was lasered at 5 weeks of age, although his feeding difficulties persisted after this procedure. His CVT was managed with casting, open reduction surgeries, pinning, and Ponseti boots. An alternating esotropia was noted at 4 months of age. He was diagnosed with dysphagia and aspiration when transitioning to solids. A barium study at 1 year of age revealed one episode of silent aspiration of thin liquids. At 13 months of age, the proband was able to roll over in both directions and sit with support. He could say “mam,” “dad,” and “gaga” and responded to his name when called. He was smiling and was very interactive with his parents. A more formal assessment showed gross motor skills at a level consistent with 3–6 months of age, fine motor skills at 4 months of age, cognitive skills at 3 months of age, and language skills and social–emotional skills at 4 months of age. The proband received early intervention with physical therapy, occupational therapy, and speech therapy. Dysphagia and chronic cough were investigated with esophagogastroduodenoscopy and bronchoscopy at 15 months of age, and mild bronchitis, possibly due to aspiration or gastroesophageal reflux disease (GERD), was diagnosed on clinical appearance, although histopathology was normal. A sleep study conducted at 15 months of age showed mild-to-moderate obstructive sleep apnea. Other medical concerns included torticollis, a left parieto-occipital dermoid cyst of the scalp that was excised, recurrent otitis media treated with placement of ear tubes, hypermetropia, and food allergies.

**Figure 1:**
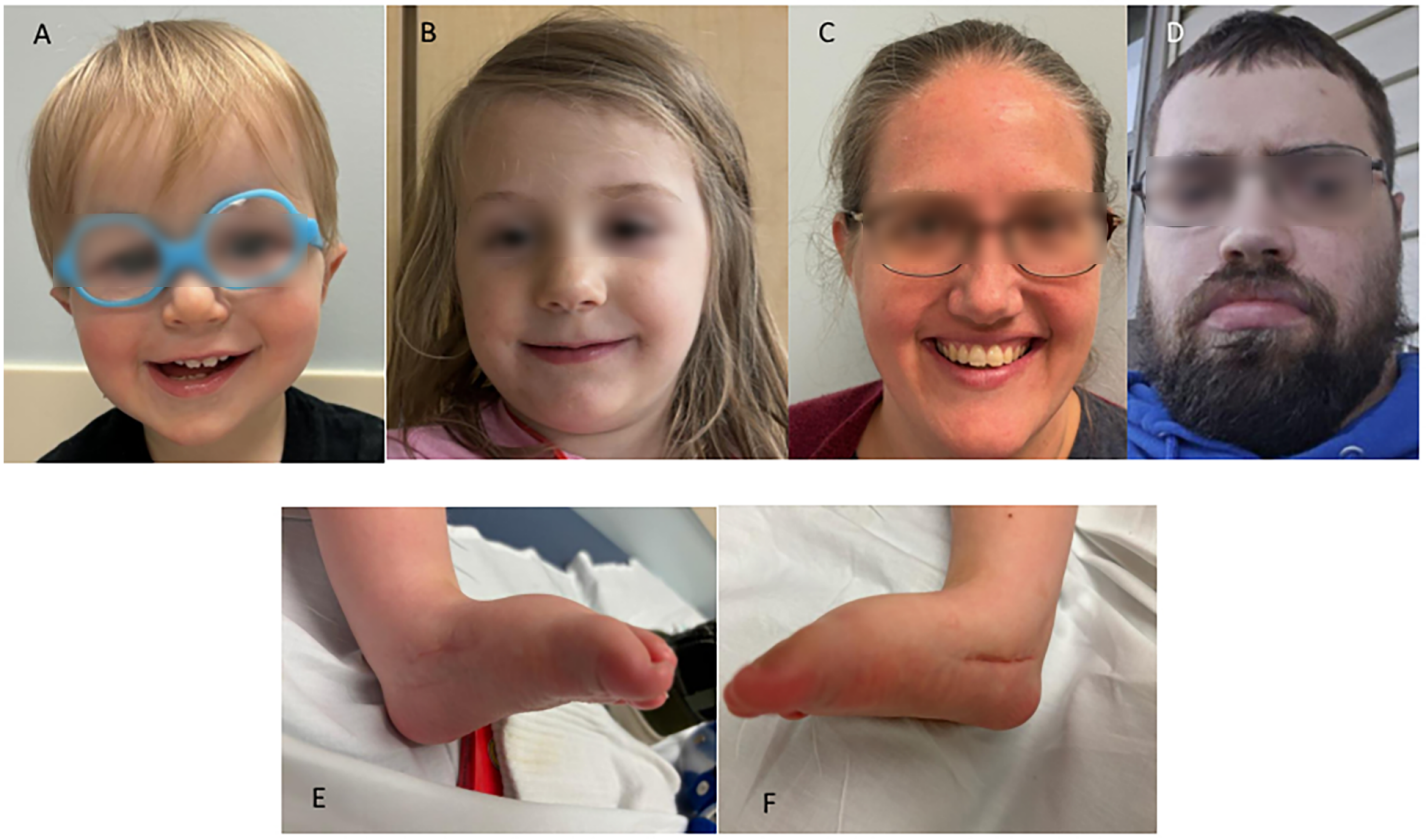
Photographs of family members with a missense variant, NM_018684.4:c.196C>T p.(Leu66Phe), in *ZC4H2*. **(A)** Facial photograph of the proband, showing a high anterior hairline and forehead, deep-set eyes, and mildly low-set ears. **(B)** Facial photograph of the proband's sister, showing mild bilateral ptosis. **(C)** Facial photograph of the proband's mother, showing mild left ptosis. **(D)** Facial photograph of the proband's maternal uncle, showing strabismus. **(E)** Photograph of the proband's left foot after surgery for congenital vertical talus. **(F)** Photograph of the proband's right foot after surgery for congenital vertical talus.

On examination at 15 months of age (Figure 1), weight was 8.74 kg (6th centile, *Z* score −1.57), and length was 73.7 cm (first centile; *Z* score −2.27). At 13 months of age, the OFC was 46.5 cm (48th centile, *Z* score −0.06). He had a high anterior hairline and forehead, deep-set eyes, mildly low-set ears with pointed ear helices, adducted thumbs, cutis marmorata, and foot deformities consistent with CVT. There were no joint contractures or ophthalmological findings besides the alternating esotropia. Brain magnetic resonance imaging (MRI) with spectroscopy revealed mild ventricular enlargement and subarachnoid fluid spaces over cerebral convexities. Suspected left frontal periventricular gray matter heterotopia was also observed, but no additional brain anomalies were detected. MR spectroscopy results were normal. The liver ultrasound results were normal.

**Figure 2:**
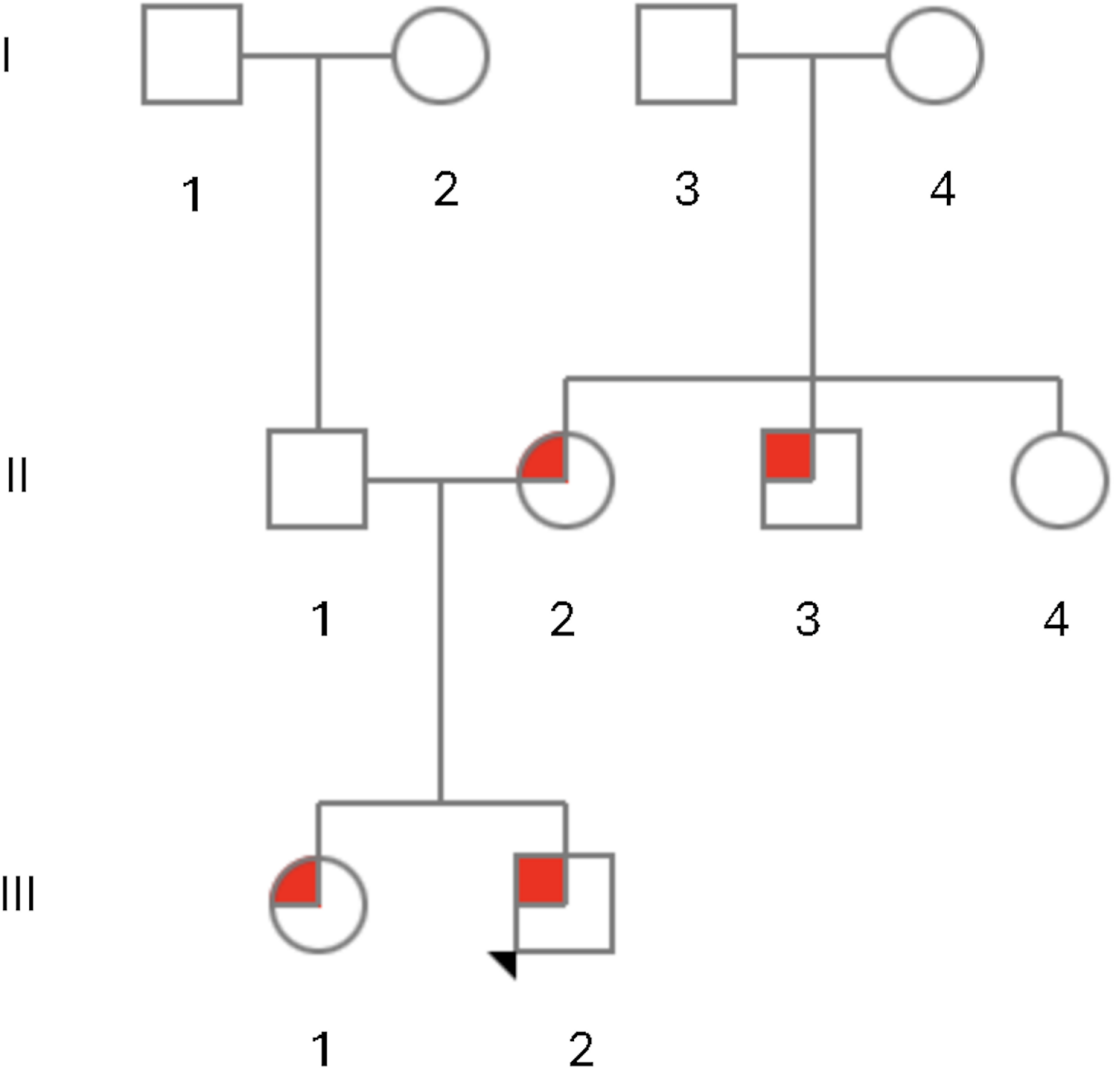
Pedigree of family members with a missense variant, NM_018684.4:c.196C>T p.(Leu66Phe), in *ZC4H2* with ZC4H2-related X-linked syndromic intellectual disability (ZARD). The proband, III-2, is indicated by the arrow and had congenital vertical talus, esotropia, and developmental delays and was hemizygous for the p.(Leu66Phe) variant in *ZC4H2*. His sister, III-1, developed tight Achilles tendons and walked on her toes and was heterozygous for the p.(Leu66Phe) variant in *ZC4H2*. His mother, II-2, had fifth finger camptodactyly and febrile seizures during childhood and was also heterozygous for the p.(Leu66Phe) variant in *ZC4H2*. The maternal uncle, II-3, was diagnosed with spastic paraplegia and cerebral palsy and had a scoliosis that required surgery and was hemizygous for the p.(Leu66Phe) variant in *ZC4H2*. The maternal aunt, II-4, reportedly had “finger abnormalities, strabismus and seizures,” but did not undergo genetic testing. The maternal grandaunt, I-4, reportedly had “cerebral palsy and was non-verbal with autistic-like features,” but did not undergo genetic testing.

In the family (Figure 2), the mother had a miscarriage at 10 weeks of gestation in her first pregnancy. The proband has one sister who was 3 years of age at the time of reporting. The sister had a tight Achilles tendon and walked on her toes. The proband's mother was 34 years of age and had bilateral camptodactyly of the fifth fingers, febrile seizures during childhood, and hypertension since the birth of the proband. She had a brother (proband's maternal uncle) who did not sit or roll on time and first walked “on tiptoes” at 3.5 years of age. He was diagnosed with cerebral palsy early in life. He had “tight” hamstrings and underwent surgery. He also had “tight” ankles, and required orthotics and had braces in high school. At 17 years of age, he was diagnosed with thoracic scoliosis of 24° that rapidly progressed to 48° and then 64° over a few months. He was treated with spinal rod insertion. Other medical concerns for the maternal uncle included strabismus that was surgically treated, a single seizure episode of unknown etiology, hypokyphosis, equinovalgus foot deformity, a myxomatous mitral valve with trivial prolapse and trivial to mild regurgitation, and anxiety. His history included physical therapy and early intervention for developmental delays. He had an individualized education plan in school and had a reduced classwork load and teacher aide until his second to last year of school but was able to graduate. At 33 years of age, he was independent in his activities of daily living; however, he did not communicate much and lived with his parents. He was his own guardian and made his own medical decisions. A limited examination by Telehealth did not show facial anomalies but was positive for shoulder asymmetry and mild thoracic scoliosis.

The maternal aunt (11-4) had strabismus that was surgically treated, seizures, and “finger abnormalities” by report, but did not undergo genetic testing. The maternal grandmother (I-4) had three male and three female siblings, including a brother with symptoms similar to those of her son described above. This individual was diagnosed with cerebral palsy and had not developed words, although he can vocalize, point, and sign. He has had behavioral challenges with “autistic-like” features. He was able to walk with a limp but has had bunions. He has had a history of gastrointestinal issues and diabetes mellitus. He lives in a group home. Other maternal relatives may also have had features of ZARD (see pedigree) but could not be seen or examined. The proband's father was 37 years of age and had bilateral clubfoot treated with special shoes and braces as a child. The paternal family history was otherwise negative.

### Genetic testing

Genetic testing with a skeletal dysplasia panel (Discover Dysplasia's Panel, Invitae) that did not contain the *ZC4H2* gene yielded negative results in the proband. Exome sequencing was performed as a trio with both biological parents at 3 months of age as a clinical test at the Genetics and Genomics Diagnostic Laboratory at Cincinnati Children's Hospital. The testing was performed in the absence of a clinical diagnosis and used the human phenotype ontology (HPO) terms—bilateral CVT, pointed ear helices, adducted thumbs, and cutis marmorata. Sequencing revealed a hemizygous, missense variant in the *ZC4H2* gene, ChrX(GRCh37):g.64141726G>A NM_018684.4:c.196C>T NP_061154.1:p.(Leu66Phe), that was maternally inherited. This variant has not been reported in the genome aggregation database (gnomAD), the 1000 genomes, the Exome Sequencing Project, dbSNP, or ClinVar databases. p.(Leu66Phe) has not been reported in the medical literature. *In silico* predictions were consistent with a potentially damaging effect for the p.(Leu66Phe) variant (Supplementary Table 1). A missense variant affecting the same amino acid but resulting in a different change, c.197T>A p.(Leu66His), has been reported to segregate with disease in a family with ZARD (5). This variant was reported in the Human Genome Mutation Database and ClinVar as pathogenic (CM159348; rs1057520297; ClinVar Variation ID: 378042). Sanger sequencing for the variant was performed in the maternal uncle and sister of the proband and both had the same, c.196C>T p.(Leu66Phe) variant, leading to the reclassification of the variant as likely pathogenic (PM5, PP1_moderate, PM2_supporting, and PP3, with two moderate criteria and two supporting criteria based on the updated ACMG criteria from ClinGen Sequence Variant Interpretation Working Group (https://clinicalgenome.org/working-groups/sequence-variant-interpretation/); (13). Determining the inheritance of the *ZC4H2* variant with Sanger sequencing does not rule out a different cause for the phenotype in these individuals that could have been identified by broader sequencing. The proband's maternal aunt and great maternal aunt did not undergo genetic testing but also have symptoms that could be attributed to the *ZC4H2* variant. The proband's exome results also showed a heterozygous, likely pathogenic variant, c.6552dup, in *HERC2* (NM_004667.5) that was paternally inherited. Biallelic variants in this gene have been associated with an autosomal recessive intellectual disability syndrome that is associated with severe neurocognitive delays, seizures, and structural brain malformations (14). However, the phenotype associated with *HERC2* variants was not present in this family. A microarray was not performed after a diagnosis was obtained with exome sequencing.

## Discussion

In 1987, Wieacker et al. linked a rare disorder characterized by congenital contractures, neuropathic muscle atrophy, cranial nerve involvement, oculomotor apraxia, dyspraxia of the facial and tongue muscles, and intellectual disability to the DXYS1 locus in proximal Xq (15). After further mapping and a candidate gene approach, the causative gene for this condition was identified as *ZC4H2* (16, 17). Pathogenic variants of *ZC4H2* can cause a variety of phenotypes, including arthrogryposis multiplex congenita (AMC) with joint flexion contractures and fetal hypokinesia/akinesia, distal limb muscle atrophy, hypotonia, motor delays, intellectual disability, and progressive brain atrophy (3–12, 18). Short stature and microcephaly, facial anomalies with a high forehead, deep-set eyes with up-slanting palpebral fissures, low-set and posteriorly rotated ears, a flat philtrum and retro-micrognathia and a short neck, sleep apnea, arrhythmia, and hypoglycemia have been noted (19–23). Although the condition predominantly affects males, ZARD has also been described in females with AMC and Pierre–Robin sequence, arthrogryposis congenita, muscular weakness, ptosis, strabismus, and oculomotor apraxia (6, 24–29). The variable phenotypes observed in female patients with deleterious *ZC4H2* variants were initially attributed to skewed X-inactivation (3), but later studies have not found a correlation between the degree of X-inactivation and the disease phenotype (5, 8, 24).

In this family, the proband was initially referred for CVT, a rare but previously described skeletal finding in ZARD (23). Rocker-bottom feet and clubfoot/equinovarus deformity have also been observed as prominent features of the prenatal and neonatal presentations of ZARD and are found in 72% and 76% of affected males, respectively (12). The fifth finger camptodactyly observed in the index patient's mother is also a recognized feature (12, 22) and is consistent with her status as a carrier of the causative variant. Other musculoskeletal findings in ZARD have included kyphosis, scoliosis, hip dislocation in addition to arthrogryposis, and flexion contractures (12), and it is noteworthy that the maternal uncle in this family had a significant scoliosis that required surgical repair. One distinctive finding in the index patient was the marked swallowing difficulty which seemed severe compared to other patients with ZARD.

This family adds to the reports of four other families with ZARD who have missense variants in the coiled-coil domain with a total of 16 males described (Supplementary Table 1; 2, 3, 5, 12). In the original family reported with ZARD, the six affected males from three generations had arthrogryposis with slowly progressive distal muscle atrophy that affected the eyes, face, tongue, and hands, severe atrophy of the distal muscles of the arms and legs, apraxia of the oculomotor muscles with ptosis and bilateral, divergent strabismus, and skeletal findings with clubfoot, pes calcaneovalgus, and scoliosis (2). Developmental delays that affected motor and speech development and dysarthria were also present (2). It was noteworthy that the condition was said to be able to be diagnosed at birth due to clubfoot and oculomotor apraxia in that family. A pedigree of four affected males and five females was first described by Miles and Carpenter (30) and later found to have c.197T>A p.(Leu66His) in *ZC4H2* (5), a missense variant that affects the same amino acid residue as the family in this report. The affected males in this family had motor delays and intellectual disabilities, spasticity/hyperreflexia, hypotonia, camptodactyly, clubfoot and other joint contractures, scoliosis, rocker-bottom feet, flat feet, facial anomalies, and exotropia (5). The p.(Leu66His) variant was only able to partially restore *gad1* expression in *zc4h2*-null zebrafish, suggesting that the variant results in reduced function (5). Lastly, two non-identical, male twins both had club feet at birth, poor feeding with reduced weight gain, reduced spontaneous movements, and developmental delays at 6 months of age [family 1 from (12)]. Both twins sat at 2 years of age and were able to mobilize with a frame from 5 years of age, although they later became wheelchair bound (12). The boys developed distal arthrogryposis with flexion contractures of the knees, and one had recurrent hip subluxation that was surgically treated (12). Both boys received speech and language therapies and one had dysarthria, but their cognitive skills were thought to be comparatively good (12). Sequencing as part of the Deciphering Developmental Disorders study (family 263304) showed c.210C>A p.(His70Gln) in *ZC4H2* (12).

It remains to be seen if missense variants in the coiled-coil domain will prove to be clinically distinguishable from other variant types in *ZC4H2*. Although all of the patients had motor delays and intellectual disability, few of the patients with missense variants in the coiled-coil domain developed seizures (1/14; 7%) compared to 22/70 (39%) in a group of male and female patients with ZARD with all types of causative variants combined and 21/42 males with all types of causative variants combined (12; Supplementary Table 1). However, this and similar comparisons are limited by the inclusion of patients with missense variants in both groups. Although none of the patients with the missense variants in the coiled-coil domain were reported to have neonatal respiratory distress, limitation of shoulder movement, hip contractures, short limbs, proximal placement of the thumbs, or edema of the dorsum of the hands and feet (Supplementary Table 1), the absence of these features in published papers may not exclude them. The family in this report had good survival, as the maternal uncle was still alive at the age of 33 years of age and the great uncle was even older, apparently without significant respiratory or cardiac concerns and without recurrent seizures, although we were unable to perform genetic testing in this individual. Other missense variants affecting different protein domains of *ZC4H2* that may be hypomorphic variants have also been associated with a relatively good prognosis (23). In contrast, male lethality has been noted with frameshift variants involving *ZC4H2*, although not all studies have found a clear phenotype–genotype correlation (12). It is interesting that the first family reported by Wieacker et al. (2) had characteristic eye findings with oculomotor apraxia, ptosis, and divergent strabismus, but these findings were not present in the remaining two families and are not unique to patients with variants involving the coiled-coil domain. The non-specific MRI finding of ventriculomegaly in the index patient has also been seen in others with ZARD (12); MRI data from other patients with missense variants in the coiled-coil domain are limited.

The ZC4H2 protein interacts with the cytosolic N-terminus of TRPV4 and positively modulates TRPV4 (31), a channel involved in various physiological processes and associated with inherited diseases. Functional studies for p.(Val63Leu), a missense variant that is located in the coiled-coil domain spanning residues 11–104 in humans, showed weaker Smad-stabilizing activity and Smad transactivation for the variant protein compared to wild-type ZC4H2 protein (4). In addition, the variant ZC4H2 proteins showed weaker interaction with Smad1 and Smad5 in co-IP assays with impaired ability to prevent Smad1/5 ubiquitination (4). It is possible that the weakened Smads-stabilizing activity of the variant ZC4H2 could be relevant to the impaired neural development in these patients (4). In studies using mouse models and cell lines, ZC4H2 was shown to be stabilized independently by both RNF220 and RLIM, the ubiquitin E3 ligase RING finger LIM domain-binding protein, and that the RLIM-ZC4H2/RNF220-Shh complex was important for cerebellar granule neuron progenitor development (32). These findings may have relevance for the neurological deficits seen in patients with ZARD. The Leu66 residue is conserved among different species and down to zebrafish (12) and is also situated in the CC domain of the protein. As coiled-coil regions frequently form homo- or heterodimers with other proteins containing coil regions, it is possible that the missense substitutions affecting residue 66 alter binding to one of the numerous predicted interactors for this gene. The milder phenotypes may be due to reduced interactions for this domain with other critical proteins, with less adverse effects compared to loss of interaction. It is also possible that at least some of the variants are hypomorphic alleles, as the p.(Leu66His) variant only partially restored *gad1* expression in the *zc4h2*-null zebrafish (5), although predictions from the AlphaMissense Pathogenicity Heatmap (33) show that all of these missense variants have a high likelihood of pathogenicity (Supplementary Table 1). Functional studies on p.(Leu66Phe) to see whether it retains partial residual ZC4H2 function, like p.(Leu66His), are needed to further understand the disease mechanism of this variant.

## Conclusion

We report a family of four individuals with ZARD caused by a missense variant in the coiled-coil domain of *ZC4H2*, NM_018684.4:c.196C>T p.(Leu66Phe). The proband presented with CVT and hypotonia and developed esotropia, motor and speech delays, and short stature. His phenotype and that of other family members was relatively mild, consistent with other missense variants in this domain, which to date have been associated with large pedigrees and the absence of significant cardiopulmonary complications.

## Data Availability

The original contributions presented in the study are included in the article/Supplementary Material, further inquiries can be directed to the corresponding author.

## Supplementary material

Supplementary Table 1 **Phenotypic and genotypic features of patients with missense variants involving the coiled-coil domain in ZC4H2**. Phenotypic features and frequencies were compared with Table 1 (12). The table shows previously reported variants (3, 5, 12) and the c.196C>T p.(Leu66Phe), in ZC4H2 from this report.
